# A MYB‐Related Transcriptional Module Cooperatively Represses Xylem Development in Poplar

**DOI:** 10.1111/pbi.70745

**Published:** 2026-08-21

**Authors:** Gaofeng Fan, Jiahui Jiang, Zihan Cheng, Feixiang Ma, Zhanling Sun, Zelin Li, Yu Qi, Guanzheng Qu, Tingbo Jiang

**Affiliations:** ^1^ State Key Laboratory of Tree Genetics and Breeding Northeast Forestry University Harbin China; ^2^ College of Horticulture and Landscape Architecture Yangtze University Jingzhou China

**Keywords:** leaf morphogenesis, *PagMYBR028*, poplar, transcriptional regulation, wood formation

## Abstract

MYB transcription factors play crucial roles in regulating plant growth and development, but the functions of the MYB‐related subfamily in woody plants remain poorly understood. In this study, we characterized *PagMYBR028*, an MYB‐related transcription factor from poplar ‘84 K’ ( *Populus alba* × *Populus glandulosa*), which was predominantly expressed in leaves and xylem. Compared with WT plants, *PagMYBR028*‐overexpressing transgenic poplar exhibited reduced leaf area and retarded secondary xylem development, whereas *PagMYBR028*‐RNAi lines displayed the opposite phenotypes. Further analysis revealed that PagMYBR028 interacts with PagMYBR005, a related regulator with overlapping functions in poplar development. Both proteins directly repress the expression of *PagGRF12b*, and their interaction enhances this transcriptional repression. In addition, overexpression of *PagGRF12b* promoted secondary cell wall deposition. Taken together, our results delineate a PagMYBR028‐PagMYBR005‐PagGRF12b module that coordinately regulates poplar growth and secondary wall development, providing new insights into the transcriptional network underlying wood formation and a theoretical basis for molecular breeding in trees.

## 1 Introduction

Wood, the most abundant renewable natural resource and industrial raw material, plays a vital role in the global carbon cycle and is widely used in fields such as papermaking and construction (Austin and Ballaré 2010; Harris et al. 2021). Wood formation originates from the division, expansion and differentiation of vascular cambium cells (Zhong and Ye 2015). Through repeated periclinal divisions, cambial stem cells differentiate inward into xylem and outward into phloem, driving the radial thickening of tree trunks (Larson 2012). Newly formed xylem cells undergo cell expansion, secondary cell wall (SCW) deposition, lignification and programmed cell death, eventually maturing into functional wood tissue (Plomion et al. 2001). The structural integrity of wood arises primarily from the biosynthesis and deposition of cellulose, hemicellulose and lignin, which together form a thickened SCW (Zhang, Chen, et al. 2024). The mechanical strength and utility of wood are largely determined by its cellular architecture and the spatiotemporal regulation of SCW deposition (Donaldson 2001). Consequently, elucidating the molecular regulatory network controlling SCW biosynthesis has become a central research focus in efforts to genetically improve wood quality and yield (Groover 2005).

The xylem consists mainly of fibres, vessel elements and ray cells. Wood fibres provide mechanical support, vessel elements enable longitudinal transport of water and solutes, and ray cells mediate radial nutrient exchange and storage (Plomion et al. 2001; Donaldson 2001). The relative proportional differentiation and spatial organization of these cell types collectively determine wood structure and properties (Rathgeber et al. 2016). Decades of research have established that lignin biosynthesis is precisely regulated by a multi‐tiered transcriptional network (Zhong and Ye 2015; Demura and Fukuda 2007; Ohtani and Demura 2019). NAC‐family transcription factors serve as the primary master regulators of this network (Kim et al. 2022). For example, VASCULAR‐RELATED NAC DOMAIN (*VND*) proteins, *VND6* and *VND7* are specifically expressed in vessel elements and regulate metaxylem and protoxylem differentiation, respectively (Yamaguchi et al. 2011). Similarly, NAC SECONDARY WALL THICKENING PROMOTING FACTOR1 (*NST1*) and SECONDARY WALL‐ASSOCIATED NAC DOMAIN PROTEIN1 (*SND1*) act as central regulators of SCW biosynthesis in fibres (Mitsuda et al. 2007). The second‐tier regulatory network is primarily composed of MYB transcription factors, which fine‐tune lignin content, composition and deposition patterns by directly regulating the expression of lignin biosynthetic enzyme genes (Xiao et al. 2021). For instance, a group of confirmed MYB transcription factors, including *MYB20*, *MYB46*, *MYB58*, *MYB63*, *MYB69*, *MYB83* and *MYB85*, act as major regulators that coordinately control secondary wall development (McCarthy et al. 2010; Zhong et al. 2008).

Beyond the core NAC‐MYB module, other transcription factors, such as WRKY, ERF and GRF families, also contribute to the precise regulation of lignin biosynthesis (Schluttenhofer and Yuan 2015; Vahala et al. 2013). These factors interact with the NAC‐MYB network to ensure spatiotemporally controlled expression of lignin‐related genes during wood formation (Demura and Fukuda 2007). For example, the *PtrMYB074*‐*PtrWRKY19* dimer directly activates downstream target *PtrbHLH186*, thereby promoting secondary xylem development (Liu et al. 2022). Conversely, *PagERF110*, highly expressed in vascular tissues, binds to the GCC‐box *cis*‐element in the *PagXND1d* promoter to repress its expression, leading to negative regulation of xylem deposition (Cheng et al. 2024). Growth‐regulating factors (GRFs), another class of plant‐specific transcription factors, also play vital roles in leaf development and secondary growth (Omidbakhshfard et al. 2015). Among the 19 GRF members identified in poplar, *PagGRF7a*, *PagGRF12a*, *PagGRF12b* and *PagGRF15* have been closely associated with leaf‐size control (Wang et al. 2020; Zhou et al. 2019). In addition to its role in leaf development, *PagGRF12a* interacts with *PagGIF1b* to suppress *PagXND1a* expression, thereby negatively regulating secondary xylem formation (Wang et al. 2021). Conversely, *PagGRF11* promotes xylem development by binding to the GARE *cis*‐element and activating *CCCH39* expression (Tian et al. 2022). Furthermore, the *PtoTCP20*‐*miR396d*‐*PtoGRF15* signalling cascade has been shown to modulate secondary vascular development (Wang et al. 2023).

MYB transcription factors are classified into four major subfamilies based on the number of MYB repeats (R) in their DNA‐binding domain: R2R3‐MYB (2R), R1R2R3‐MYB (3R), 4R‐MYB and MYB‐related (1R) (Dubos et al. 2010). Research on MYB transcription factors involved in SCW development has primarily focused on the R2R3‐MYB subfamily (Nakano et al. 2015). In contrast, despite being the second‐largest subgroup, the MYB‐related subfamily remains underexplored. To address this research gap, we analysed the expression profiles of MYB‐related genes during secondary growth in poplar ‘84 K’. By integrating transcriptomic, phenotypic and physiological analyses, we identified a regulatory network centred on *PagMYBR028* and established a functional PagMYBR028‐PagMYBR005‐PagGRF12b module. Within this module, the *PagMYBR028*‐*PagMYBR005* form a dimer that directly represses the transcription of *PagGRF12b*, influencing leaf morphogenesis and negatively regulating SCW deposition in poplar. Our results uncover a previously uncharacterized regulatory network that controls SCW synthesis, providing a theoretical framework for understanding tree growth mechanisms and facilitating targeted genetic improvement of wood properties.

## 2 Results

### 2.1 *PagMYBR028* Is Predominantly Expressed in Leaves and Stems and Regulates Poplar Growth

**FIGURE 1:**
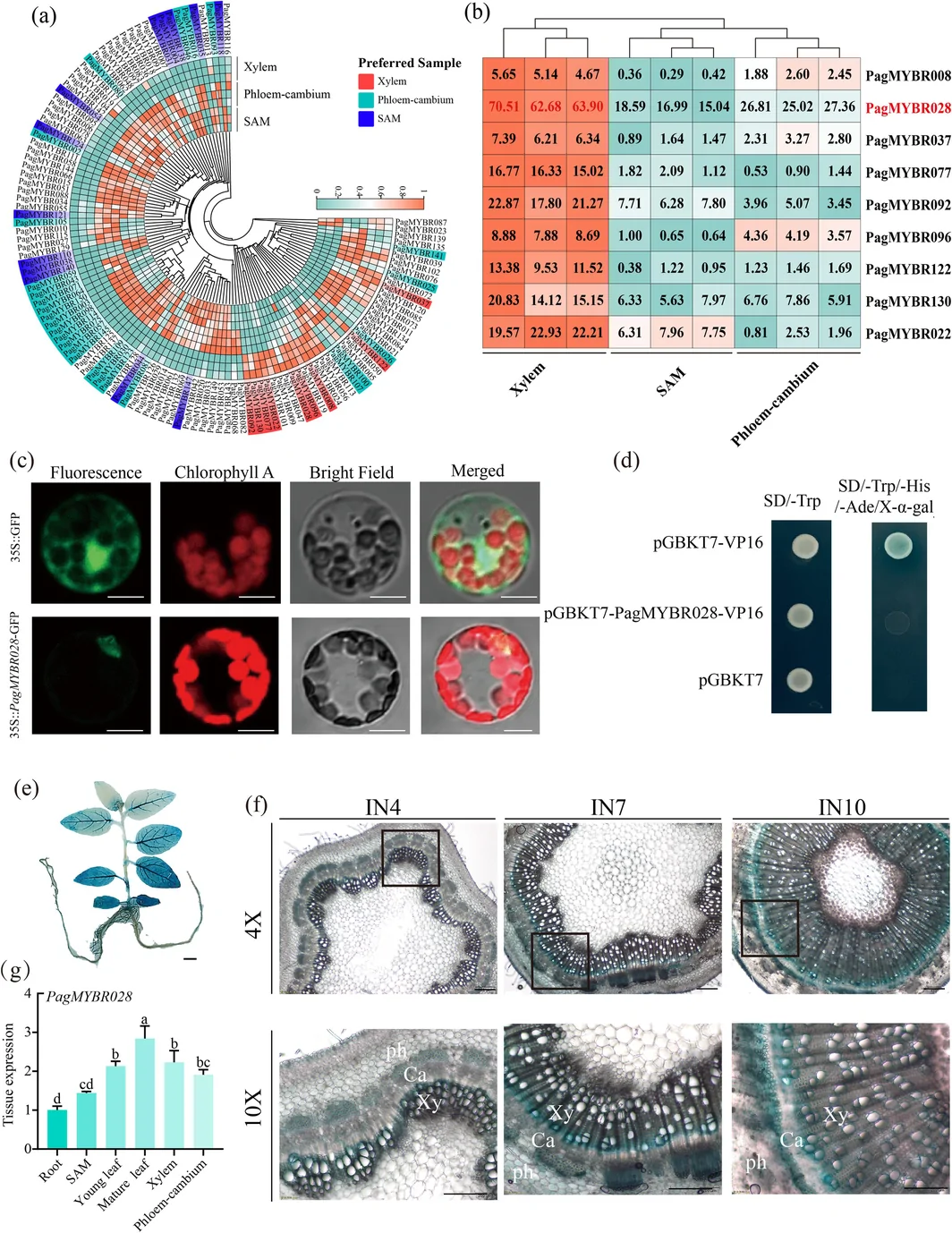
Expression profiling and molecular characterization analysis of *PagMYBR028* transcription factor in poplar. (a) Transcriptome profiling of MYB‐related transcription factors in various tissues of poplar ‘84 K’. (b) Expression patterns of xylem‐preferential MYB‐related genes. (c) Subcellular localization of PagMYBR028:GFP fusion protein in poplar leaf protoplasts; scale bar = 10 μm. (d) The transcriptional self‐activation assay of PagMYBR028 protein. (e) Histochemical GUS staining of Pro*PagMYBR028*::GUS transgenic poplar; scale bar = 1 cm. (f) GUS staining of stem vascular tissues at the 4th, 7th, and 10th internodes. ph, phloem; Ca, cambium; Xy, xylem; scale bars: 4×, 200 μm; 10×, 100 μm. (g) RT‐qPCR analysis of *PagMYBR028* expression across different poplar tissues. Data are presented as mean ± SD (*n* = 3). Statistically significant differences were determined by one‐way ANOVA method.

Among 152 MYB‐related members identified in poplar, 136 genes were detected in shoot apical meristem (SAM), phloem‐cambium, or xylem of poplar ‘84 K’ based on transcriptome data. After excluding 16 genes with FPKM values below 1, the expression analysis of the remaining 120 genes showed *PagMYBR035* exhibited the highest τ value (0.817) and high expression level in the SAM, whereas *PagMYBR083* showed the lowest τ value (0.110) (Table S1). Using a threshold of τ > 0.5, we identified 26, 9 and 13 genes predominantly expressed in the phloem‐cambium, xylem and SAM, respectively (Figure 1a). Notably, *PagMYBR028* showed the highest transcript abundance in xylem (Figure 1b), suggesting its potential role in wood development.

Sequence analysis showed that PagMYBR028 protein encoded 338 amino acids containing a typical SANT domain, sharing 97.92% and 62.30% amino acid sequence identity with its orthologs PtrMYBR028 in *Populus trichocarpa* and KUODA1 in *Arabidopsis thaliana* , respectively (Figure S1). Transient expression assays in poplar leaf protoplasts demonstrated that PagMYBR028‐GFP fusion protein was exclusively localized to the nucleus, whereas the GFP control was distributed uniformly throughout the cell (Figure 1c). In addition, yeast strains carrying PagMYBR028‐VP16 and pGBKT7 exhibited markedly inhibited growth on selective SD/−Trp/−His/−Ade medium, while the positive control pGBKT7‐VP16 grew normally and developed a blue coloration in the presence of X‐α‐gal (Figure 1d). These results indicate that PagMYBR028 is a nuclear‐localized protein lacking transcription self‐activation activity. Subsequently, the expression pattern of *PagMYBR028* was also determined, and GUS staining driven by its promoter revealed strong activity in SAM, young leaves, mature leaves, stems and roots (Figure 1e). Furthermore, histochemical analysis of stem cross‐sections from the 4th, 7th and 10th internodes showed that GUS signals were predominantly localized in xylem cells of secondary vascular tissues, with weaker signals observed in the phloem‐cambium (Figure 1f). Tissue‐specific RT‐qPCR further supported this expression profile (Figure 1g), suggesting an important role of *PagMYBR028* in wood formation.

**FIGURE 2:**
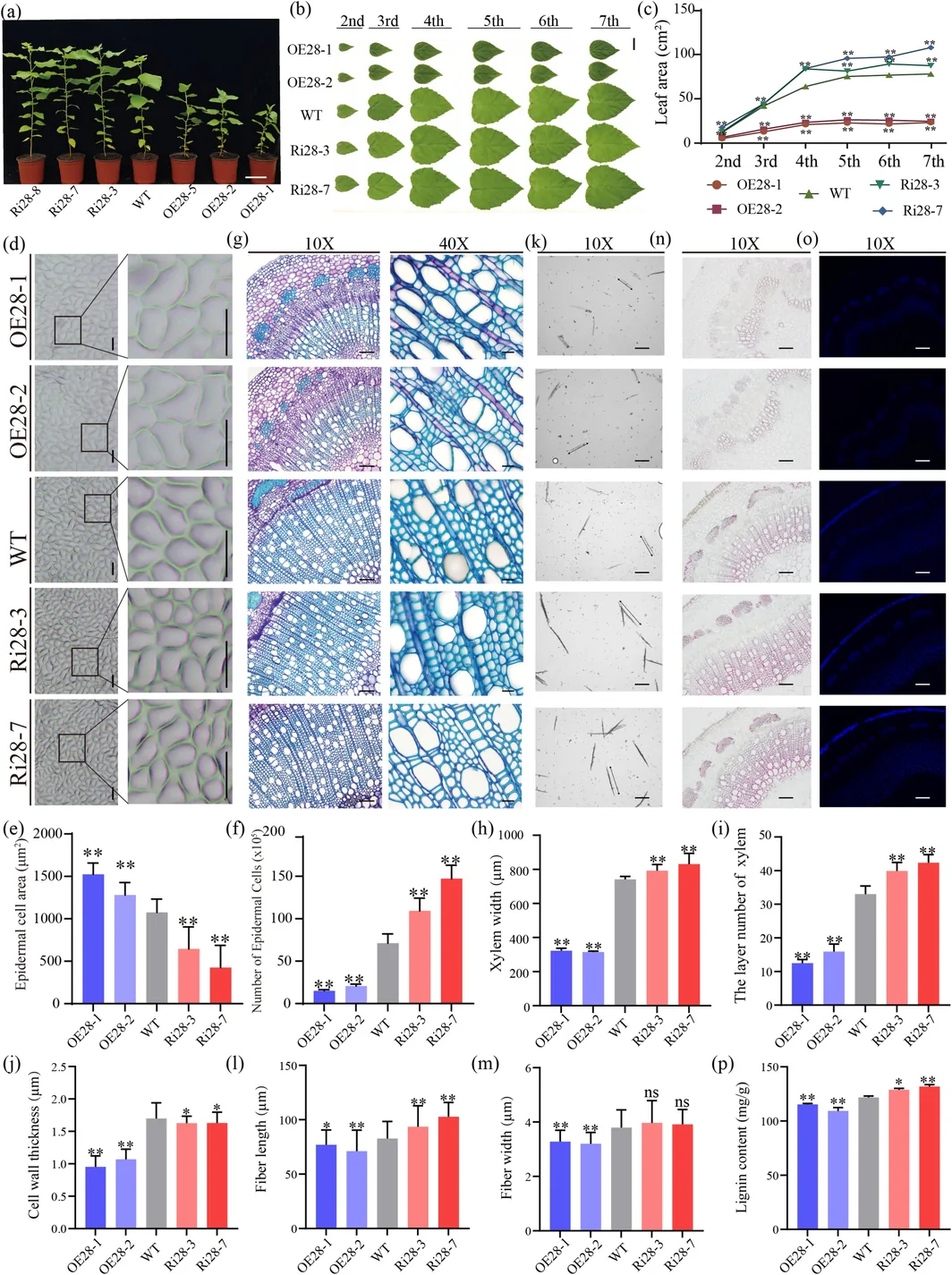
*PagMYBR028* regulates poplar leaf and stem development. (a) Phenotypes of WT, *PagMYBR028*‐overexpression (OE), and *PagMYBR028*‐RNAi (Ri) lines. Scale bar = 10 cm. (b) Representative leaf observation from internodes 2nd to 7th of the corresponding lines. Scale bar = 2 cm. (c) Leaf area. (d) Observation of epidermal cells in the fifth leaf. Scale bar = 50 μm. (e) Epidermal cell area. (f) Epidermal cell number. (g) Cross‐sectional images of the 14th internode xylem in WT and *PagMYBR028*‐transgenic lines. (h) Xylem width. (i) Xylem cell layers. (j) Xylem cell wall thickness. (k) Fibre cell morphology. (l) Fibre length. (m) Fibre width. (n) Lignin staining by phloroglucinol‐HCl. (o) Lignin autofluorescence. Scale bar of g, k, n, o: 10×, 100 μm; 40×, 20 μm. (p) Total lignin content. Data are presented as mea*n* ± SD (*n* ≥ 3). Asterisks indicate significant differences from WT (Student's *t*‐test; *p < 0.05, **p < 0.01).

To investigate the function of *PagMYBR028* in poplar growth and development, we generated both overexpression (OE) and RNA interference (RNAi) transgenic lines. Two overexpression lines (OE28‐1 and OE28‐2) and two RNAi lines (Ri28‐3 and Ri28‐7) showing significant alterations in expression level were selected based on RT‐qPCR analysis (Figure S2). After 3 months of greenhouse cultivation, *PagMYBR028*‐OE lines exhibited significantly reduced plant height, ground diameter and leaf area, but a significantly increased axillary bud number compared to WT. Conversely, *PagMYBR028*‐RNAi lines showed increased plant height and leaf area, with no significant change in stem diameter (Figure S3; Figure 2a,b).

### 2.2 *PagMYBR028* Affects Poplar Leaf Morphogenesis

Observation of the leaves of *PagMYBR028* transgenic poplar revealed marked morphological differences compared to WT. The leaf area of *PagMYBR028*‐OE lines was significantly reduced by 45.3%–71.8% across 2nd‐7th leaves, whereas *PagMYBR028*‐RNAi lines exhibited an increase of 5.27%–49.0% (Figure 2c). To elucidate the cytological basis underlying these morphological variations, microscopic observations of the 5th leaf epidermal cells were conducted. The results showed that the cell area of OE28‐1 and OE28‐2 was increased by 41.6% and 17.4%, respectively, while the corresponding cell number was reduced by 78.9% and 70.5% compared with WT. Conversely, the Ri28 lines displayed reduced cell area but increased cell number (Figure 2d–f). Furthermore, RT‐qPCR showed that cell expansion‐related genes *EXPA3a* and *EXPA3b* were significantly up‐regulated in the *PagMYBR028*‐OE lines, whereas the cyclin genes *CYCB1;1a* and *CYCB1;1b* were markedly down‐regulated. In contrast, the *PagMYBR028*‐RNAi lines displayed the opposite expression trends (Figure S4). Collectively, these results demonstrated that *PagMYBR028* regulates poplar leaf morphogenesis by coordinately modulating cell expansion and cell division processes.

### 2.3 *PagMYBR028* Inhibits Poplar Secondary Xylem Development

Given the significant reduction in stem diameter observed in *PagMYBR028*‐OE poplar, we further investigated its role in stem secondary growth. Cytological observations of stem cross‐sections revealed severely delayed secondary xylem development in the *PagMYBR028*‐OE lines, whereas xylem development was markedly accelerated in the *PagMYBR028*‐RNAi lines (Figure S5). Quantitative measurements of the 14th internode demonstrated that *PagMYBR028* strongly suppresses secondary xylem formation. The OE28‐1 and OE28‐2 showed a drastic reduction in xylem cell layer number (51.66% and 62.19%), secondary xylem width (56.39% and 57.17%) and cell wall thickness (37.06% and 43.96%), respectively. Conversely, the Ri28‐3 and Ri28‐7 showed an increase in xylem cell layers (20.94% and 28.28%) and secondary xylem width (6.84% and 12.17%), although a decrease in cell wall thickness (8.68% and 9.16%) was observed (Figure 2g–j). Consistently, fibre morphology analysis further revealed that OE28‐1 and OE28‐2 had significantly shorter fibre length (6.99% and 9.62%) and fibre width (13.75% and 15.72%), while the Ri28‐3 and Ri28‐7 displayed longer fibre lengths (13.10% and 23.35%) without significant change in width (Figure 2k–m).

To explore the underlying mechanism of these cell wall phenotypes, lignin deposition was analysed both qualitatively and quantitatively. Phloroglucinol‐HCl staining and lignin autofluorescence imaging showed weaker signals in *PagMYBR028*‐OE plants and stronger signals in *PagMYBR028*‐RNAi lines compared to WT (Figure 2n,o). Quantitative measurements confirmed that lignin content decreased by 5.38%–10.27% in *PagMYBR028*‐OE lines, whereas it increased by 5.73%–8.12% in *PagMYBR028*‐RNAi lines (Figure 2p). Furthermore, the expression profiling of lignin biosynthesis‐related genes demonstrated a general downregulation in *PagMYBR028*‐OE lines, while an opposite trend was observed in *PagMYBR028*‐RNAi lines (Figure S6). Collectively, these results demonstrate that *PagMYBR028* acts as a negative regulator of secondary cell wall biosynthesis in poplar.

### 2.4 PagMYBR028 Interacts With PagMYBR005

**FIGURE 3:**
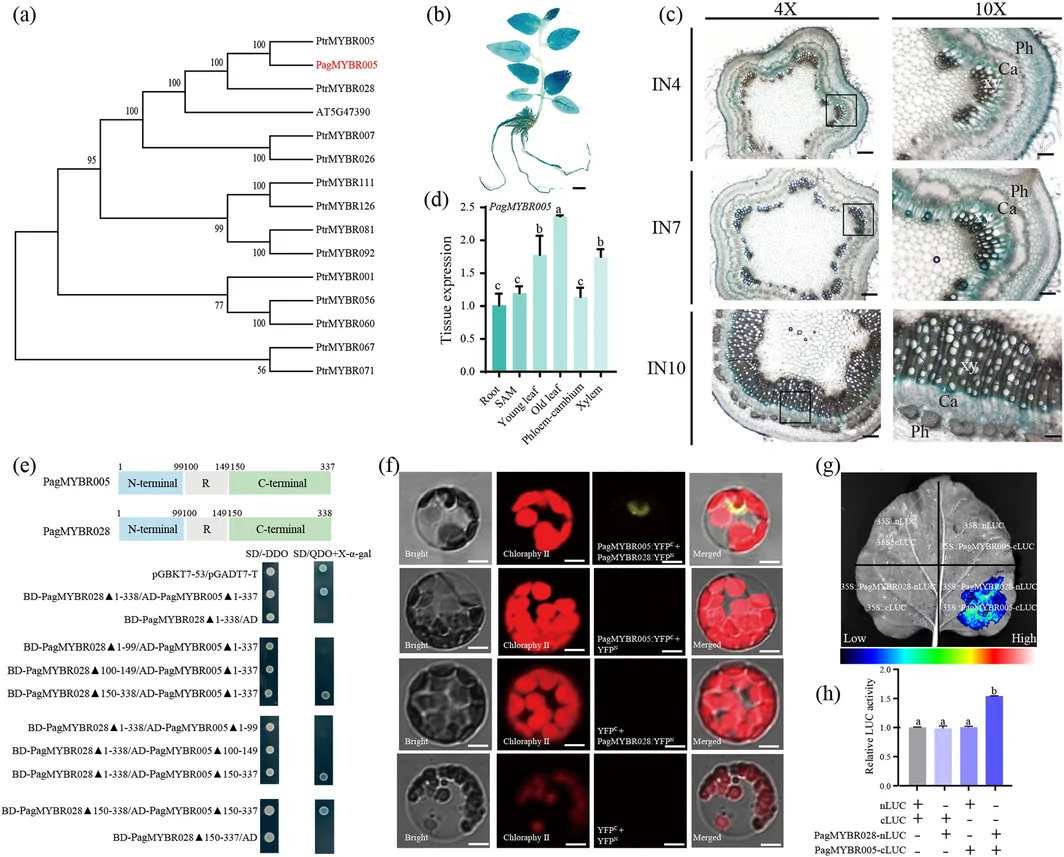
Screening of interacting proteins for PagMYBR028 and validation of protein–protein interactions. (a) Phylogenetic analysis of PagMYBR005 with MYB‐related proteins from *P. trichocarpa* . (b) Histochemical GUS staining of Pro*PagMYBR005*::GUS transgenic poplar; scale bar = 1 cm. (c) GUS staining of stem vascular tissues at the 4th, 7th, and 10th internodes. ph, phloem; Ca, cambium; Xy, xylem. Scale bars: 4×, 200 μm; 10×, 100 μm. (d) RT‐qPCR analysis of *PagMYBR005* expression across different poplar tissues. (e) Yeast two‐hybrid assay for PagMYBR028 and PagMYBR005 proteins. (f) Bimolecular fluorescence complementation assay. Scale bar = 10 μm. (g) Luciferase complementation assay. (h) Determination of LUC activity. Data are presented as mea*n* ± SD (*n* ≥ 3). Statistically significant differences were determined by one‐way ANOVA method.

To screen for interacting proteins of PagMYBR028, Y2H was conducted using PagMYBR028 protein as bait and poplar cDNA library as prey. This screen identified PagMYBR005, an MYB‐related protein, as a potential interacting partner of PagMYBR028. PagMYBR005 protein encoded 337 amino acids and shared 97.92% sequence similarity with PtrMYBR005 in *P. trichocarpa* (Figure 3a; Figure S7). Tissue expression analysis and GUS reporter assays driven by the *PagMYBR005* promoter revealed a spatial expression pattern similar to that of *PagMYBR028*, with high abundance in leaves and xylem (Figure 3b–d), suggesting possible functional coordination between the two genes.

The physical interaction between PagMYBR028 and PagMYBR005 was verified using Y2H, bimolecular fluorescence complementation (BiFC), and luciferase complementation assays (LCA). In Y2H assay, growth status on SD/‐Trp/‐His/‐Ade/‐Leu/X‐α‐gal selective media confirmed a direct interaction between PagMYBR028 (150–338 aa) and PagMYBR005 (150–337 aa) (Figure 3e). BiFC assays in poplar leaf protoplasts showed clear nuclear fluorescence (Figure 3f). Additionally, the LCA test demonstrated that co‐transfection with the two genes in tobacco resulted in significantly higher luciferase activity than other combinations (Figure 3g,h). Collectively, these results demonstrate a direct interaction between PagMYBR028 and PagMYBR005.

### 2.5 *PagMYBR005* Regulates Poplar Development

**FIGURE 4:**
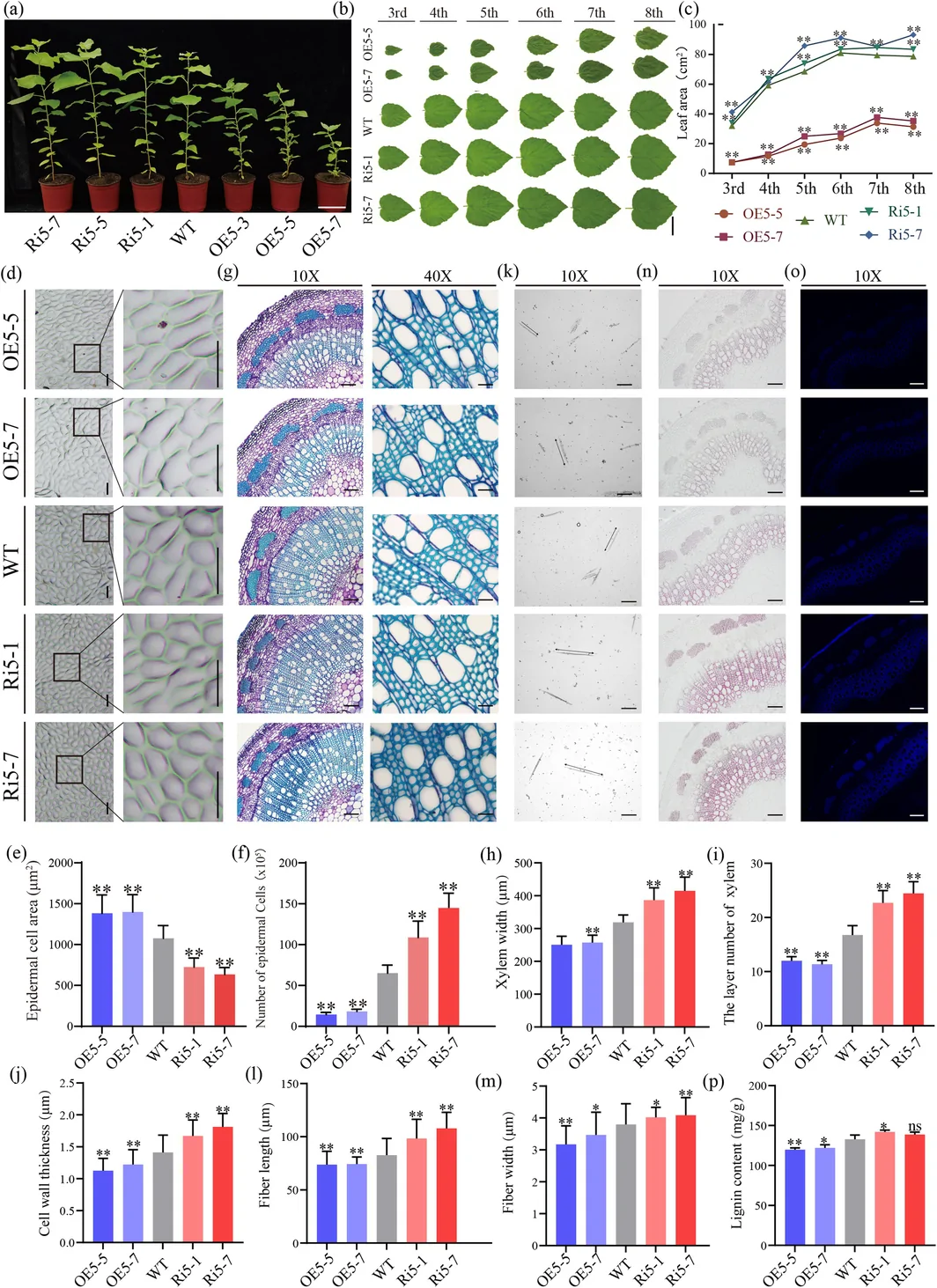
*PagMYBR005* regulates poplar leaf and stem development. (a) Phenotypes of WT, *PagMYBR005*‐overexpression (OE), and *PagMYBR005*‐RNAi (Ri) lines. Scale bar = 10 cm. (b) Representative leaf observation from internodes 2nd to 7th of the corresponding lines. Scale bar = 2 cm. (c) Leaf area. (d) Observation of epidermal cells in the fifth leaf. Scale bar = 50 μm. (e) Epidermal cell area. (f) Epidermal cell number. (g) Cross‐sectional images of the 12th internode xylem in WT and *PagMYBR005*‐transgenic lines. (h) Xylem width. (i) Xylem cell layers. (j) Xylem cell wall thickness. (k) Fibre cell morphology. (l) Fibre length. (m) Fibre width. (n) Lignin staining by phloroglucinol‐HCl. (o) Lignin autofluorescence. Scale bar of g, k, n, o: 10×, 100 μm; 40×, 20 μm. (p) Total lignin content. Data are presented as mea*n* ± SD (*n* ≥ 3). Asterisks indicate significant differences from WT (Student's *t*‐test; *p < 0.05, **p < 0.01).

To characterize the biological function of *PagMYBR005* in poplar development, 10 *PagMYBR005‐*OE and 9 *PagMYBR005‐*RNAi (Ri) transgenic lines were generated. Two independent lines from each group were chosen for detailed phenotypic analysis (Figure S8). After 3 months of growth, *PagMYBR005‐*OE lines showed significant reductions in plant height, ground diameter and leaf area compared to WT plants. In contrast, *PagMYBR005‐*RNAi lines displayed no significant difference in plant height or ground diameter, but showed an increase in leaf area (Figure 4a–c; Figure S9). Statistical analysis of the fifth leaf revealed that leaf area was reduced by 71.7% and 63.7% in OE5‐5 and OE5‐7, respectively, whereas epidermal cell area increased (28.4% and 30.0%) and cell number decreased (77.8% and 72.0%). In contrast, Ri5‐1 and Ri5‐7 exhibited increased leaf area (2.5% and 7.7%), reduced cell area (34.8% and 42.3%) and increased cell number (67.0% and 117.1%) (Figure 4d–f). Moreover, RT‐qPCR analysis revealed significant up‐regulation of *EXPA3a* and *EXPA3b* and strong down‐regulation of *CYCB1;1a* and *CYCB1;1b* in the *PagMYBR005*‐OE lines, whereas opposite expression trends were observed in the *PagMYBR005*‐RNAi lines (Figure S10), collectively supporting the role of *PagMYBR005* in leaf morphogenesis.

Anatomical observation of stem cross‐sections revealed that *PagMYBR005*‐OE lines exhibited a reduction in secondary xylem width, whereas *PagMYBR005*‐RNAi lines displayed the opposite trend (Figure S11). Quantitative analysis of the 12th internode revealed that OE5‐5 and OE5‐7 had 28.42% and 32.28% fewer xylem cell layers, 19.36% and 21.45% narrower xylem, and 13.45% and 20.21% thinner cell walls, respectively. In contrast, Ri5‐1 and Ri5‐7 displayed 35.31% and 45.71% more xylem cell layers, 18.83% and 36.91% wider xylem, and 18.32% and 28.37% increased cell wall thickness (Figure 4g–j). Fibre morphology analysis indicated that *PagMYBR005*‐OE lines produced shorter (10.20%–10.69%) and narrower (8.71%–13.48%) fibres, whereas the *PagMYBR005*‐RNAi lines showed opposite characteristics (Figure 4k–m). Consistently, phloroglucinol‐HCl staining and autofluorescence intensity indicated weaker signals in *PagMYBR005*‐OE lines and stronger signals in *PagMYBR005*‐RNAi lines (Figure 4n,o). Quantitative measurements showed reduced lignin content in *PagMYBR005*‐OE lines, while it increased in *PagMYBR005*‐RNAi lines (Figure 4p). Moreover, RT‐qPCR analysis showed that the expression of most lignin biosynthetic genes was down‐regulated in *PagMYBR005*‐OE lines but up‐regulated in *PagMYBR005*‐RNAi lines (Figure S12). Collectively, these results demonstrate that *PagMYBR005* acts as a negative regulator of secondary cell wall deposition.

### 2.6 Transcriptome and Metabolome Analysis Reveal *PagMYBR028* Is Involved in Lignin Biosynthesis

To elucidate the molecular mechanisms by which *PagMYBR028* influences xylem differentiation in poplar, integrated transcriptomic and metabolomic analyses were conducted using peeled stem nodes from WT and *PagMYBR028*‐OE plants. Transcriptome sequencing identified 6153 differentially expressed genes (DEGs), including 3393 down‐regulated and 2760 up‐regulated. KEGG enrichment analysis revealed that these DEGs were primarily involved in carbohydrate metabolism, amino acid metabolism, plant hormone signal transduction, phenylpropanoid biosynthesis and MAPK signalling (Figure S13). Metabolomic profiling detected 1077 differential metabolites, of which 427 were up‐regulated and 650 were down‐regulated (Table S2). These metabolites were mainly enriched in pathways associated with the TCA cycle, arachidonic acid metabolism, pentose phosphate pathway, phenylpropanoid biosynthesis and tryptophan metabolism (Figure S14).

**FIGURE 5:**
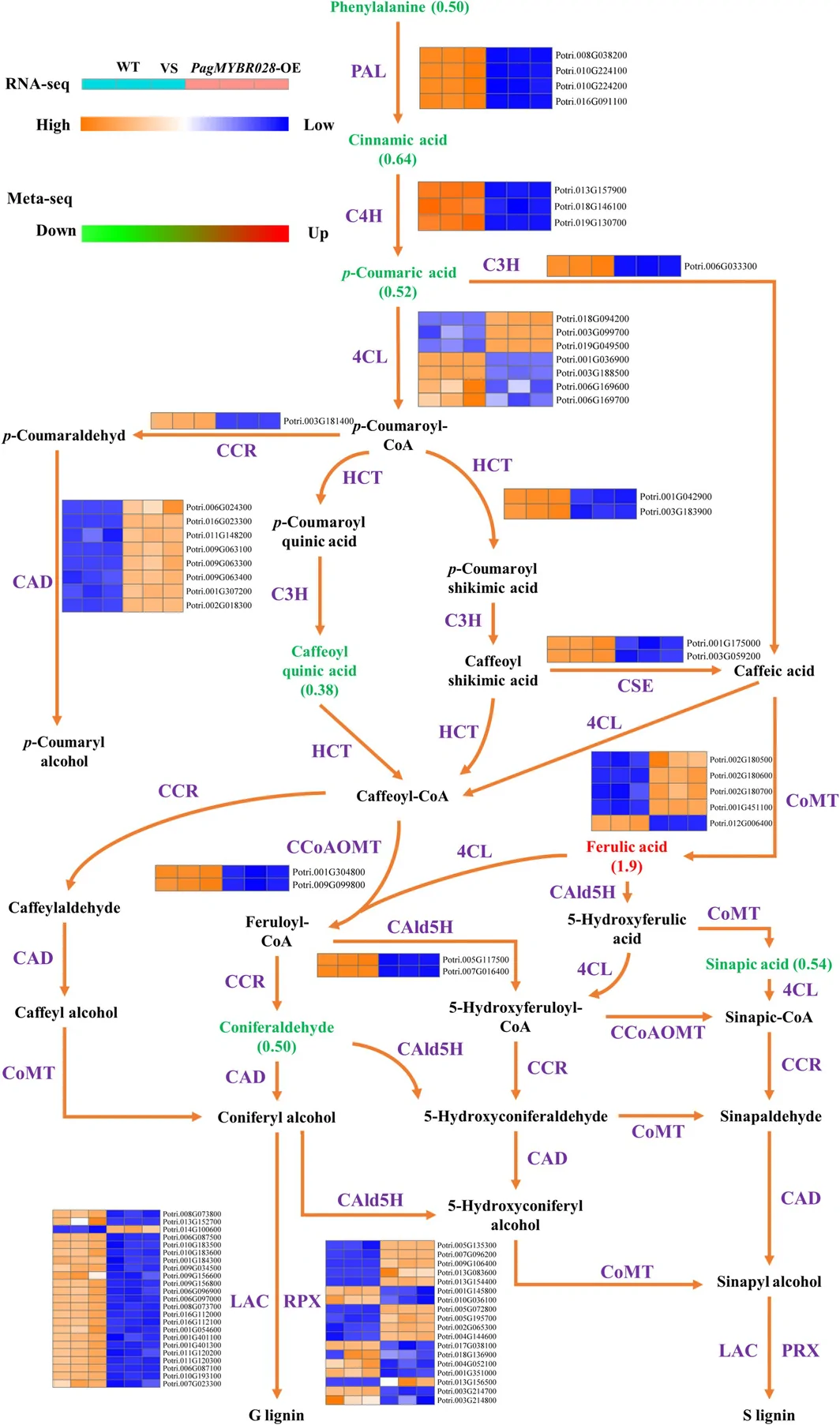
Multi‐omics profiling of the phenylpropanoid biosynthesis pathway in WT and *PagMYBR028*‐overexpressing poplar. Expression pattern analysis of differentially expressed genes and metabolites.

The integrated omics analysis revealed a profound transcriptional reprogramming of phenylpropanoid biosynthesis in *PagMYBR028*‐OE plants compared to WT. This restructuring was characterized by the dysregulation of 13 key enzyme‐encoding genes. Most pathway genes, including members of the *PAL* (4), *C3H* (1), *C4H* (3), *CCR* (1), *HCT* (3), *CSE* (2), *CCoAOMT* (2) and *CAld5H* (2) families, were suppressed, 8 *CAD* genes were markedly up‐regulated (Table S3). Consistent with these transcriptional changes, a total of 11 phenylpropanoid‐related metabolites were significantly altered. Notably, the levels of syringin, *p*‐cinnamic acid, sinapic acid, *p*‐coumaric acid, phenylalanine, coniferaldehyde and caffeoyl quinic acid were significantly reduced in *PagMYBR028*‐OE lines. By contrast, ferulic acid content was notably elevated (Figure 5).

### 2.7 Both *PagMYBR005* and *PagMYBR028* Inhibit the Expression of *PagGRF12b* Gene

**FIGURE 6:**
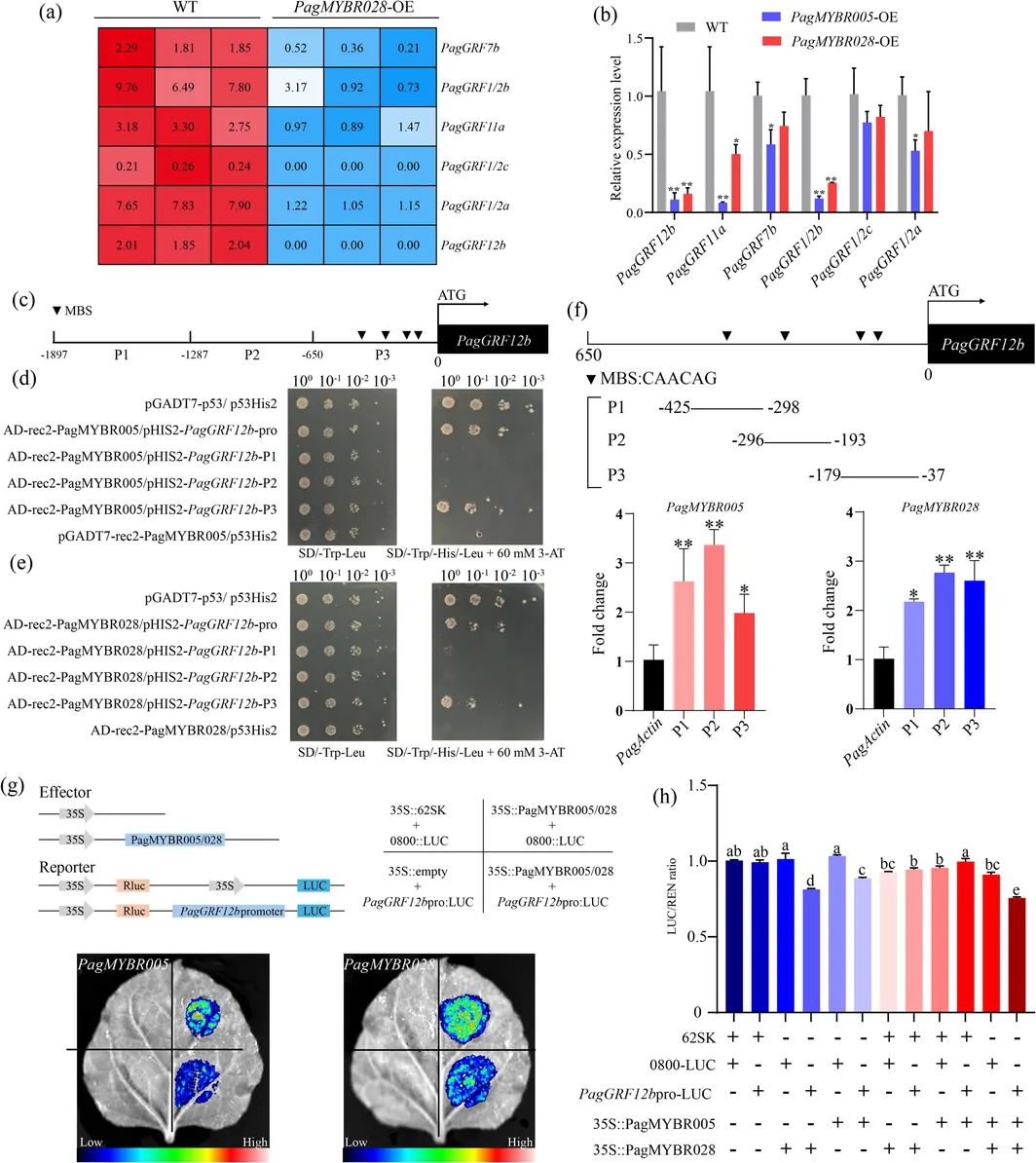
Both *PagMYBR005* and *PagMYBR028* inhibit the transcription of *PagGRF12b* gene. (a) Expression profiles of *GRF* genes in the *PagMYBR028*‐overexpression transcriptome. (b) Relative expression of *GRF* genes in *PagMYBR005*‐overexpressing lines and *PagMYBR028*‐overexpressing lines. (c) *Cis*‐acting element analysis in the *PagGRF12b* promoter. (d) Yeast one‐hybrid assay of PagMYBR005 binding to fragments of the *PagGRF12b* promoter. (e) Yeast one‐hybrid assay of PagMYBR028 binding to fragments of the *PagGRF12b* promoter. (f) ChIP‐qPCR analysis of *PagMYBR005*:3xFlag and *PagMYBR028*:3xFlag lines at the MBS regions in the *PagGRF12b* promoter. Data in (b, f) are presented as mean ± SD (*n* = 3). Asterisks indicate significant differences from control (Student's *t*‐test; *p < 0.05, **p < 0.01). (g) Dual‐luciferase assay of PagMYBR005 or PagMYBR028 binding to the *PagGRF12b* promoter. (h) Luciferase activity detection of *PagMYBR005* and *PagMYBR028* on *PagGRF12b* transcription. Statistically significant differences were determined by one‐way ANOVA method.

Transcriptome analysis revealed that six GRF family genes were significantly downregulated in *PagMYBR028*‐OE poplar (Figure 6a). RT‐qPCR analysis further confirmed that the expression of all six GRF genes was consistently suppressed in both *PagMYBR005*‐OE and *PagMYBR028*‐OE lines (Figure 6b). Among these, *PagGRF12b* has been reported to repress leaf cell expansion and enhance cell proliferation in poplar (Wang et al. 2020), implying that it may function downstream of *PagMYBR028* and *PagMYBR005*. To test this hypothesis, we carried out yeast one‐hybrid (Y187), chromatin immunoprecipitation quantitative PCR (ChIP‐qPCR) and dual‐luciferase reporter assays. The results demonstrated that both *PagMYBR005* and *PagMYBR028* specifically bound to the *PagGRF12b* promoter region (−650 to 0 bp), which contained four MYB‐binding sites (MBS: CAACAG) (Figure 6c–e). ChIP‐qPCR assays using *PagMYBR005*:3 × Flag and *PagMYBR028*:3 × Flag overexpression poplar showed significant enrichment at these MBS regions, with *PagMYBR005* showing enrichment factors of 2.6‐, 3.4‐ and 2.0‐fold, and *PagMYBR028* showing 2.2‐, 2.8‐ and 2.6‐fold enrichment relative to the control (Figure 6f). To further evaluate the effect of *PagMYBR005* or *PagMYBR028* on *PagGRF12b* transcription, we conducted transient expression assays in tobacco leaves. Co‐infiltration of tobacco leaves with *PagMYBR005* or *PagMYBR028* together with the *PagGRF12b* promoter resulted in weaker fluorescence signals and significantly reduced LUC activity (Figure 6g,h). Notably, co‐expression of both *PagMYBR005 and PagMYBR028* together with the *PagGRF12b* promoter caused a further decline in LUC activity, indicating a synergistic enhancement of transcriptional repression.

### 2.8 Overexpression of *PagGRF12b* Promotes Xylem Development

**FIGURE 7:**
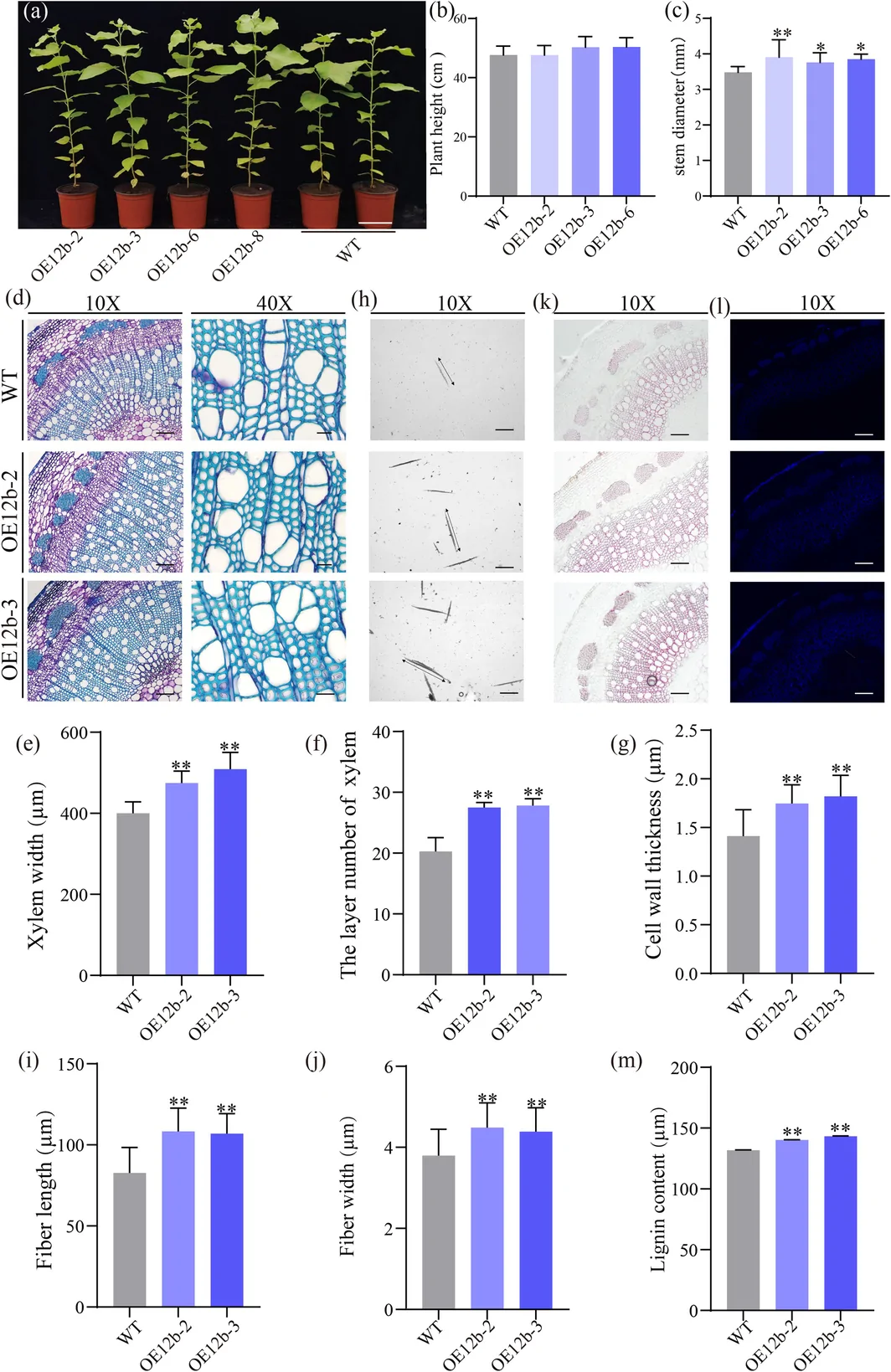
*PagGRF12b* promotes xylem development in poplar. (a) Representative images of 3‐month‐old WT and *PagGRF12b*‐overexpression (OE) lines. Scale bar = 10 cm. (b) Plant height. (c) Stem diameter. (d) Cross‐sectional images of xylem. (e) Xylem width. (f) Xylem cell layers. (g) Xylem cell wall thickness. (h) Fibre cell morphology. (i) Fibre length. (j) Fibre width. (k) Lignin staining with phloroglucinol‐HCl. (l) Lignin autofluorescence. Scale bar of d, h, k, l: 10×, 100 μm; 40×, 20 μm. (m) Total lignin content. Data are presented as mea*n* ± SD (*n* ≥ 3). Asterisks indicate significant differences from WT (Student's *t*‐test; *p < 0.05, **p < 0.01).

To elucidate the gene function of *PagGRF12b*, we generated nine *PagGRF12b*‐OE poplar lines and selected the two lines with the highest expression (OE12b‐2 and OE12b‐3) for detailed analysis (Figure S15). Phenotypic observations and statistical results revealed that *PagGRF12b*‐OE plants exhibited a significant increase in ground diameter, whereas no notable difference in plant height was observed compared to the WT (Figure 7a–c). Cross‐sectional analysis of stem internodes indicated that xylem development was significantly accelerated in *PagGRF12b*‐OE lines (Figure S16). In addition, the OE12b‐2 and OE12b‐3 exhibited increases in xylem cell layer number (35.09% and 37.04%), secondary xylem width (18.59% and 27.08%) and cell wall thickness (23.81% and 28.98%) relative to WT (Figure 7d–g). Furthermore, fibre morphology was notably affected, with fibre length and width increasing by 29.31%–31.00% and 15.55%–18.19%, respectively (Figure 7h–j). Phloroglucinol‐HCl staining and lignin autofluorescence analyses revealed stronger staining intensity and enhanced fluorescence signals in *PagGRF12b*‐OE poplar relative to WT (Figure 7k,l). Subsequent quantitative analysis further verified that lignin content was significantly higher in the *PagGRF12b*‐OE lines (Figure 7m).

## 3 Discussion

MYB transcription factors are essential for plant secondary development, cell differentiation and morphogenesis (Dubos et al. 2010; Xiao et al. 2021). However, current research have focused on the R2R3‐MYB subfamily, whereas the MYB‐related subfamily has received limited attention. In this study, we demonstrate that *PagMYBR028* and *PagMYBR005* are interacting MYB‐related transcriptional repressors that coregulate leaf and wood development. They function by repressing the transcription of *PagGRF12b*, a promoter of xylem differentiation, thereby defining a mechanism that integrates organ growth with secondary cell wall deposition.

Phylogenetic analysis placed PagMYBR028 within the same clade as KUODA1, a key regulator of leaf cell expansion in *A. thaliana* (Figure S1) (Lu et al. 2014). Notably, *PagMYBR028* exhibited a tissue‐specific expression pattern closely resembling that of *KUODA1* (Figure 1) (Huang et al. 2015), suggesting potential evolutionary conservation of function. However, the overexpression of *PagMYBR028* in poplar resulted in a reduction of leaf area and cell density, accompanied by an increase in epidermal cell size (Figure 2a–f). In contrast, overexpression of *KUODA1* promoted cell enlargement without altering cell number (Lu et al. 2014). These findings suggest that *PagMYBR028* may have acquired a new functionalized role in woody plants, regulating the balance between cell division and expansion during leaf morphogenesis. Similar species‐specific functional diversification has been observed in other gene families. For instance, *Arabidopsis* phytochrome‐interacting factors (PIFs) inhibit xylem differentiation through *CLE44* activation (Ghosh et al. 2022), whereas *PtoPIF3* promotes secondary growth by activating *PtoHB7/8* in *Populus tomentosa* (Xiao et al. 2025). Such divergence underscores the evolutionary plasticity of regulatory networks between herbaceous and woody plants.

Secondary growth in plants is governed by a hierarchical transcriptional network centered on the NAC‐MYB‐secondary wall biosynthesis module (Kim et al. 2022). In our study, the overexpression of *PagMYBR028* led to pronounced reductions in plant height, stem diameter and xylem thickness (Figure 2; Figure S3). Anatomical observations further showed that xylem differentiation was suppressed and lignin deposition was reduced in *PagMYBR028*‐OE lines, whereas *PagMYBR028*‐RNAi plants displayed the opposite phenotypes (Figure 2; Figure S5), confirming *PagMYBR028* as a negative regulator of secondary wall formation. Transcriptome profiling found that *PagMYBR028* represses key regulators within the NAC‐MYB secondary wall network, including *WND2a*/*WND2b*, *WND3B*, *WND5A*/*WND5B*, *MYB152*, *MYB092*, *MYB002*/*MYB021* and *MYB020* (Table S4). The biological relevance of these transcription factors is well established. For example, a quadruple mutant of *PtrWND1A/1B/2A/2B* exhibited impaired secondary wall deposition in xylem fibres, xylem ray parenchyma and phloem fibres, whereas overexpression of *PtrWND2B* enhanced secondary wall thickness (Zhong et al. 2010). Similarly, *PtrMYB3* and *PtrMYB20* are directly activated by the NAC master regulator *PtrWND2B*, thereby promoting the biosynthesis of cellulose, xylan and lignin (McCarthy et al. 2010). *PtoMYB092* and *PtrMYB152* enhance lignin synthesis by activating the expression of lignin pathway genes, such as *CCoAOMT1* (Li et al. 2015; Li, Wang, et al. 2014). In addition, the expression of *PAL*, *C3H*, *C4H*, *CCR*, *HCT*, *CSE*, *CCoAOMT* and *CAld5H*, all of which participate in the phenylalanine metabolic pathway, was further downregulated. Metabolomic analysis also supported this observation, revealing decreased levels of phenylalanine‐derived intermediates, including sinapic acid, *p*‐coumaric acid, phenylalanine, coniferaldehyde and caffeoyl quinic acid (Figure 5). Previous studies have shown that *PtoPAL3*, activated by *PtoNAC90*, contributes to xylem development (Sun et al. 2025). In the lignin pathway, *CCoAOMT* is critical for guaiacyl lignin biosynthesis and serves as a key precursor for syringyl lignin formation (Zhong et al. 2000). Likewise, *CAld5H* is essential for converting guaiacyl‐type into syringyl‐type lignin monomers; its overexpression elevates the S/G ratio by 64% in poplar and substantially alters lignin composition (Li et al. 2003). Collectively, these findings demonstrate that *PagMYBR028* modulates lignin biosynthesis not only by repressing the transcription of key genes but also by restricting metabolic flux through the phenylpropanoid pathway.

Transcription factors frequently regulate gene expression by interacting with other proteins to form transcriptional complexes (Filtz et al. 2014). For instance, *ONAC127* and *ONAC129* form a heterodimer that directly regulates the expression of sugar transporter genes, *OsMST6* and *OsSWEET4*, facilitating photoassimilate translocation into the endosperm during grain filling and contributing to heat stress tolerance (Ren et al. 2021). Similarly, *SlERF.B2* and *SlERF.B5* dimerize to cooperatively activate *SlGGP1* and other genes, thereby promoting ascorbic acid accumulation in tomato leaves and ripe fruits and improving seedling stress resistance (Chen et al. 2025). In contrast, *PbMYB61* binds to the *PbLAC1* promoter to promote lignin biosynthesis in pear. Meanwhile, *PbMYB308* disrupts this process by interacting with *PbMYB61* and blocking its transcriptional activity (Zhu et al. 2023). Our study demonstrated a direct physical interaction between PagMYBR005 and PagMYBR028, as confirmed by multiple independent assays (Figure 3). This interaction suggests that *PagMYBR028* may act synergistically with *PagMYBR005* to regulate secondary developmental processes. Moreover, overexpression of *PagMYBR005* resulted in reduced leaf size and impaired xylem differentiation, phenotypes that closely mirror those observed in *PagMYBR028*‐OE plants (Figure 4). This functional convergence, combined with their physical interaction, suggested that *PagMYBR005* and *PagMYBR028* act as negative regulators in leaf and xylem development, likely through the assembly of a shared transcriptional repressor complex.

**FIGURE 8:**
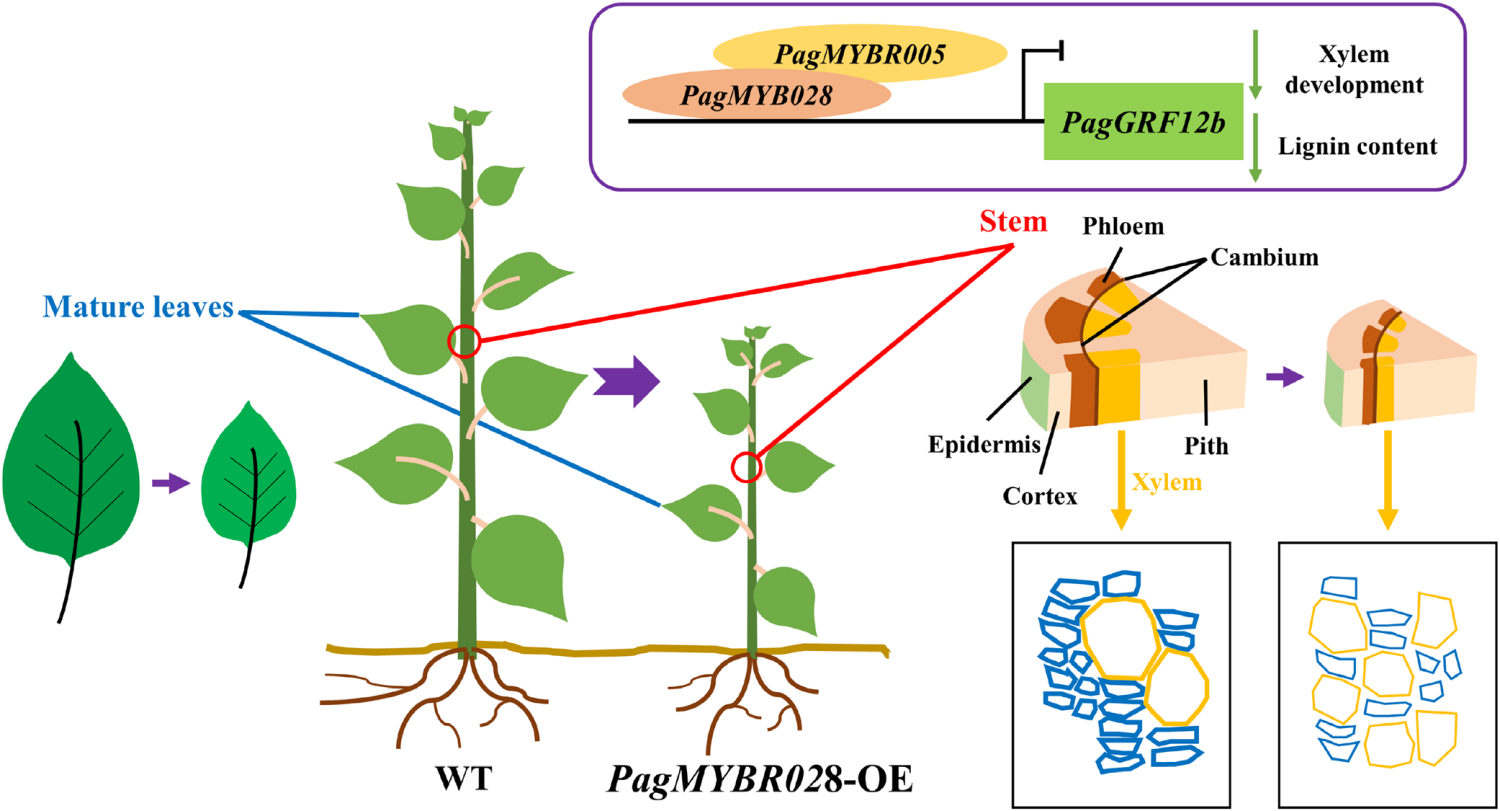
The PagMYBR028–PagMYBR005–PagGRF12b model regulates wood formation in poplar.

GRFs are plant‐specific transcription factors that play pivotal roles in modulating plant growth and organ development (Omidbakhshfard et al. 2015). In our study, transcriptomics revealed that *PagGRF12b* was downregulated in the *PagMYBR028*‐overexpression line (Figure 6a). Previous research showed that overexpression of *PagGRF12b* increases leaf area by reducing cell size while increasing cell number (Wang et al. 2020). We hypothesized that *PagMYBR028* directly targets *PagGRF12b*, and this was confirmed by yeast one‐hybrid, luciferase reporter and ChIP‐qPCR assays, which demonstrated a transcriptional repression relationship between *PagMYBR028* and *PagGRF12b* (Figure 6c–g). Functional validation further revealed that *PagGRF12b*‐overexpression poplar promoted xylem differentiation and lignin accumulation (Figure 7), supporting its function as a positive regulator of secondary growth. Notably, our results demonstrate that *PagMYBR005* also binds to the promoter of *PagGRF12b* and represses its expression, and its interaction with *PagMYBR028* synergistically strengthens this inhibition (Figure 6h), suggesting that MYB proteins may function within integrated transcriptional networks to balance secondary wall deposition. This regulatory pattern is similar to that reported in other plants. For instance, *PuMYB40* and *PuWRKY75* not only individually activate their target genes, *PuLRP1* and *PuERF003*, to promote adventitious rooting in *Populus ussuriensis*, but also form a heterodimer that synergistically enhances this transcriptional activation, thereby accelerating this process (Wang et al. 2022). Similarly, the dimerization of the *MYB2*/*MYB108*‐*LBD29* transcription complex stimulates lateral root development by activating the expression of *CDEF1* in *Arabidopsis* (Zhang et al. 2024). Collectively, these findings highlight a novel layer of MYB‐GRF regulatory interaction in poplar wood formation, where *PagMYBR005* and *PagMYBR028* synergistically repress *PagGRF12b* to negatively regulate xylem differentiation (Figure 8).

## 4 Experimental Procedures

### 4.1 Plant Materials and Growth Conditions

Poplar ‘84 K’ (*Populus alba × P.* *glandulosa*) and tobacco (*Nicotiana benthamiana*) plants were cultivated under greenhouse conditions with a 16 h light/8 h dark photoperiod, relative humidity of 65%–75% and temperature set at 24°C ± 2°C. Sterile poplar tissues were propagated on half‐strength MS (1/2 MS) medium supplemented with 25 g/L sucrose, 0.05 mg/L indole‐3‐butyric acid (IBA), 0.02 mg/L naphthaleneacetic acid (NAA) and 6 g/L agar. After 1 month of in vitro propagation, tissue‐cultured seedlings were transplanted into greenhouse soil and grown for an additional 3 months for phenotypic and growth analyses. Phenotypic observations and statistical analyses of growth‐related indicators were conducted on healthy three‐month‐old plants.

### 4.2 Gene Cloning, Vector Construction and Poplar Transformation

Full‐length CDS of *PagMYBR005*, *PagMYBR028* and *PagGRF12b*, along with their approximately 2000 bp promoter regions, were cloned from the poplar ‘84 K’ by polymerase chain reaction (PCR). To construct promoter‐reporter vectors, the promoter sequences of *PagMYBR005* and *PagMYBR028* were used to replace the *Cauliflower Mosaic Virus* 35S (CaMV 35S) promoter in the pBI121 vector, thereby driving the expression of the β‐glucuronidase (GUS) reporter gene. The open reading frames (ORFs) of these three genes were individually inserted into the pBI121‐3xFlag vector under the control of the 35S promoter. For RNA interference (RNAi) constructs, specific 200‐bp fragments targeting *PagMYBR005* and *PagMYBR028* were designed based on the NCBI database (https://www.ncbi.nlm.nih.gov/) and cloned in sense and antisense orientations into the pFGC5941 expression vector. Genetic transformation of poplar was performed using the *Agrobacterium tumefaciens* strain GV3101 via the leaf disc transformation method, following the method of Fan et al. (Fan et al. 2025). Transgenic lines were verified by PCR and reverse transcription‐quantitative PCR (RT‐qPCR) to confirm gene integration and expression (Regier and Frey 2010). The specific primers designed are listed in Table S5.

### 4.3 Tissue Expression Pattern Analysis of MYB‐Related Proteins

Total RNA was extracted from various tissues of two‐month‐old poplar ‘84 K’ using the RNA Sample Prep Kit (Cat. No. 74104), including shoot apical meristem (SAM), phloem‐cambium and xylem, with three biological replicates per tissue. RNA purification, quality assessment and transcriptome sequencing followed Wang et al. (Wang, Pak, et al. 2022), and expression was quantified as FPKM. MYB family members were identified by searching the *P. trichocarpa* genome with HMMER 3.1 using the MYB DNA‐binding domain (Pfam PF00249). Candidates were validated via NCBI‐CDD (https://www.ncbi.nlm.nih.gov/Structure/cdd/wrpsb.cgi) and Pfam (https://www.ebi.ac.uk/interpro/entry/pfam/), and those containing a single R repeat were retained. The resulting genes were named by chromosomal locations (Yang et al. 2021). Tissue specificity of gene expression was assessed using the τ coefficient, ranging from 0 (broad expression) to 1 (high tissue specificity), calculated as described by Yanai et al. (Yanai et al. 2005).

To validate the expression of *PagMYBR005* and *PagMYBR028*, tissues were collected from two‐month‐old poplar, including young leaves (1st to 3rd leaves), mature leaves (9th to 12th leaves), shoot apical meristem, phloem‐cambium, xylem and roots. Total RNA was isolated using the RNAisoPlus reagent (TaKaRa, Dalian, China) and cDNA was synthesized with the PrimeScript RT reagent Kit with gDNA Eraser (TaKaRa, Dalian, China) following the manufacturer's instructions. RT‐qPCR was performed using an Applied Biosystems 7500 real‐time PCR system with TB Green Premix Ex Taq II FAST qPCR kit (TaKaRa, Dalian, China). *PagActin* served as the internal reference gene, and gene relative expression levels were calculated using the 2^−ΔΔCt^ method. Each experiment was performed with three biological replicates and three technical replicates.

### 4.4 Subcellular Localization

The coding sequence of *PagMYBR028* was cloned into the pUC19:GFP vector to generate the pUC19‐PagMYBR028:GFP construct. Poplar protoplasts were isolated using a modified method based on a published protoplast system (Abel and Theologis 1994). Subsequently, the recombinant plasmid pUC19‐PagMYBR028:GFP or the empty pUC19:GFP control was introduced into the protoplasts via PEG‐mediated transformation. After 12 h of incubation in darkness at room temperature, GFP fluorescence was visualized using a laser scanning confocal microscope (LSM800 with Airyscan).

### 4.5 Statistics of Leaf Area, Epidermal Cell Area and Number

To evaluate leaf morphological variation between WT and transgenic poplar, fully expanded leaves at defined developmental stages were collected and digitally imaged. Leaf area was measured using ImageJ software (v1.8.0_322) (https://imagej.net/ij/), with three biological replicates per genotype. Prior to measurement, images were calibrated using the built‐in scale bar, and leaf areas were obtained by tracing leaf margins with the freehand selection tool and executing the Measure command. Abaxial epidermal impressions were also prepared using the nail‐polish peeling technique and visualized under a Zeiss microscope. Epidermal cell area and density were quantified with ImageJ by manually outlining individual cell boundaries and recording area values. Cell density was then calculated as the number of cells per mm^2^. To ensure statistical robustness, at least 100 epidermal cells were examined per biological replicate.

### 4.6 Histological and Histochemical Analysis

To analyse gene expression patterns and histological structures in poplar stems, a series of staining and microscopic observations were conducted. Stem nodes from two‐month‐old plants were immersed in GUS staining solution (containing 0.2 M PBS, 0.05 M K_3_[Fe(CN)_6_], 0.5 M K_4_[Fe(CN)_6_], 0.5 M EDTA, 0.2% Triton X‐100 and 0.6% X‐Gluc). After vacuum infiltration to ensure complete tissue immersion, the samples were incubated overnight at 37°C in the dark. Subsequently, tissues were decolorized in a solution of absolute ethanol and glacial acetic acid (3:1, v/v), and GUS activity was visualized under a Zeiss microscope.

For cytological analysis, internode segments from three‐month‐old poplar plants were fixed in 50% FAA solution and dehydrated through a graded ethanol series (50%, 60%, 70%, 80%, 95% and 100%). The tissues were then infiltrated with a xylene‐paraffin mixture (3:1) and embedded in pure paraffin. Sections of 10 μm thickness were obtained using a rotary microtome, stained with 0.01% toluidine blue, and digitally imaged with the Zeiss microscope. To visualize lignin distribution, sections were treated with 1% phloroglucinol‐HCl solution and immediately observed under a microscope. Additionally, lignin autofluorescence in cross‐sections of the same stem segments was captured using an upright fluorescence microscope equipped for lignin‐specific fluorescence channel.

### 4.7 Analysis of Cell Wall Composition

To analyse fibre cell morphology, stem segments from different materials were debarked, sectioned into small sections and immersed in a maceration solution composed of 10% chromic acid and 10% nitric acid (1:1, v/v) for 1 h. After thorough washing with deionized water, the fibre length and width were observed and measured under a Zeiss microscope, with at least 100 cells counted per material. Additionally, debarked stem tissues collected below the 12th internode from three‐month‐old WT and transgenic plants were collected, dried at 95°C, ground into fine powder and subjected to lignin content determination using lignin content assay kit (AKSU010U, Boxbio). All data were obtained from three independent biological replicates.

### 4.8 Yeast Two‐Hybrid Assay

To identify potential proteins interacting with PagMYBR028, yeast two‐hybrid (Y2H) assay was performed. The CDS region of *PagMYBR028* was cloned into the pGBKT7‐VP16 vector to construct pGBKT7‐PagMYBR028‐VP16, which was subsequently transformed into Y2H‐Gold yeast cells. The empty pGBKT7‐VP16 vector served as a positive control, and the pGBKT7 empty vector was used as a negative control. Transformed yeast cells were cultured on SD/‐Trp and on SD/‐Trp/‐His/‐Ade/X‐α‐gal medium, followed by incubation at 30°C for 3 days to monitor growth.

After confirming that PagMYBR028 lacked self‐activation activity, its CDS was cloned into the pGBKT7 vector as a bait protein (bait) for library screening against yeast two‐hybrid cDNA library derived from poplar ‘84 K’. Primary screening for interacting proteins was carried out on SD/‐Trp/‐His/‐Ade/‐Leu medium. Positive clones that grew were subjected to plasmid extraction and sequencing, and homology analysis was performed using BLASTp in the NCBI database.

For further verification of protein–protein interaction, full‐length and various coding regions of *PagMYBR028* and *PagMYBR005* were constructed into pGBKT7 and pGADT7 vectors, respectively. Following the Matchmaker Gold Yeast Two‐Hybrid System Kit (Clontech, USA) protocol, the recombinant plasmids were co‐transformed into the Y2H‐Gold yeast cell using the Yeastmaker Yeast Transformation System 2 (Takara). Co‐transformants were inoculated on SD/‐Trp/‐Leu medium and tested for interaction on SD/‐Trp/‐His/‐Ade/‐Leu/X‐α‐gal. The pGBKT7‐53/pGADT7‐T pair served as a positive control, while pGBKT7‐PagMYBR028/pGADT7 pair served as a negative control.

### 4.9 Bimolecular Fluorescence Complementation Assay (BiFC)

The CDS regions of *PagMYBR005* and *PagMYBR028* were cloned into the pUC‐CEYFP and pUC‐NEYFP vectors, respectively, to generate the fusion constructs PagMYBR005::YFP^C^ and PagMYBR028::YFP^N^. Poplar leaf protoplasts were isolated and co‐transformed with PagMYBR00*5*::YFP^C^ and PagMYBR028::YFP^N^ plasmids via PEG‐mediated transformation (Abel and Theologis 1994), along with three control combinations: PagMYBR005::YFP^C^ + YFP^N^, YFP^C^ + PagMYBR028::YFP^N^ and YFP^C^ + YFP^N^. Following transformation, protoplasts were cultured for 12 h in the dark. Yellow fluorescent protein (YFP) complementation signals were subsequently observed and captured using the laser scanning confocal microscope (LSM800 with Airyscan).

### 4.10 Luciferase Complementation (LCA) Assay

The full‐length region of *PagMYBR005* and *PagMYBR028* was cloned into the pCAMBIA1300‐cLUC and pCAMBIA1300‐nLUC vectors, respectively. Each plasmid was individually transformed into *Agrobacterium* strain GV3101 (pSoup). Subsequently, equal volumes of four bacterial combinations (PagMYBR005‐cLUC/PagMYBR028‐nLUC, PagMYBR005‐cLUC/nLUC, cLUC/PagMYBR028‐nLUC and cLUC/nLUC) were infiltrated into the abaxial surface of one‐month‐old tobacco leaves. Then the plants were maintained under greenhouse conditions for 2–3 days. Leaves from the infiltrated zones were subsequently excised and evenly coated with 1 mg/mL D‐luciferin potassium salt solution (CL6930, Coolaber). Following a 15‐min incubation, luciferase luminescence was detected and imaged using a Tanon‐5200 live‐cell fluorescence imaging system (Shanghai, China). Quantitative LUC activity was measured using the Luciferase Assay System (E1500, Promega) according to the manufacturer's instructions, with three biological replicates and three technical replicates for each reaction.

### 4.11 Transcriptome Sequencing

To compare the transcriptional profiles between WT and *PagMYBR028*‐OE poplar, debarked stem tissues from the 12th to 20th internodes of three‐month‐old greenhouse‐grown plants were collected, with three biological replicates per genotype. RNA sequencing was performed commercially by Genewiz Biological Technology Co. Ltd. using the Illumina platform. Raw sequencing data were assessed for quality using FastQC (v0.12.0), and low‐quality reads were trimmed and filtered using Trimmomatic (v0.40) to obtain high‐quality clean data (Wingett and Andrews 2018; Bolger et al. 2014). The clean reads were then aligned to the *Populus trichocarpa* v3.1 (https://phytozome‐next.jgi.doe.gov/) reference genome using HISAT2 (v2.2.1), and transcript abundances were quantified with StringTie (v2.2.1) (Shibaguchi and Yasutaka 2022; Pertea et al. 2015). Differential expression analysis was performed using the DESeq2 (v1.30.0) package in R, with genes meeting the thresholds of |log_2_(fold change)| ≥ 1 and *padj* ≤ 0.01 (Love et al. 2014). Gene Ontology (GO) functional annotation and Kyoto Encyclopedia of Genes and Genomes (KEGG) pathway enrichment analyses of the differentially expressed genes were conducted using the clusterProfiler package (v4.20.0) (Wu et al. 2021).

### 4.12 Metabolome Sequencing

Metabolomic profiling was conducted using liquid chromatography–tandem mass spectrometry (LC–MS/MS) (Want et al. 2006). Metabolite extraction was performed with 80% methanol as the extraction solvent (Sellick et al. 2011). Chromatographic separation was performed on a Vanquish ultra‐high performance liquid chromatography (UHPLC) system (Thermo Fisher Scientific, Germany) and was detected using an Orbitrap Q Exactive HF high‐resolution mass spectrometer (Thermo Fisher Scientific, Germany). The full‐scan mass range was set from *m/z* 100 to 1500. Raw data were processed using Compound Discoverer 3.1 software for peak alignment, identification and quantitation. Peak intensities were normalized by total spectral intensity. Metabolite identification and quantification were performed by querying the mzCloud, mzVault and MassList databases. Subsequently, the identified metabolites were annotated using the KEGG databases. Differentially metabolites were screened based on the following criteria: variable importance in projection (VIP) ≥ 1, *p* < 0.05, and fold change (FC) ≥ 1.2 or ≤ 0.833.

### 4.13 Yeast One Hybrid Analysis (Y187)

The full‐length coding sequences of *PagMYBR005* and *PagMYBR028* were cloned into the AD‐rec2 vector (Clontech, USA) containing GAL4 activation domain to generate prey constructs. Meanwhile, the full‐length promoter region of *PagGRF12b*, along with specific fragments, was inserted into the pHIS2 reporter vector (Clontech, USA) to serve as bait. Then prey and bait constructs were co‐transformed into Y187 yeast cells using the Y187‐pHis2 system Yeast One‐Hybrid Interaction Verification Kit (YH1011, Coolaber). The co‐transformation of pGADT7‐p53/p53His2 served as the positive control, while AD‐rec2‐PagMYBR005/p53His2 or AD‐rec2‐PagMYBR028/p53His2 were used as negative controls. Transformed yeast cells were plated on SD/‐Trp/‐Leu or SD/‐Trp/‐His/‐Leu with 60 mM 3‐amino‐1,2,4‐triazole (3‐AT; Sigma‐Aldrich) medium. The growth status of yeast colonies was then examined to assess potential protein‐DNA interactions.

### 4.14 Chromatin Immunoprecipitation Assay

The chromatin immunoprecipitation (ChIP) experimental procedure was adapted from previously published methods with minor modifications (Li, Lin, et al. 2014). Briefly, fresh tissue samples (~3 g) were cross‐linked by vacuum infiltration with 1% formaldehyde for 45 min, and the reaction was quenched with 3 M glycine. Samples were flash‐frozen in liquid nitrogen and ground into a fine powder. Nuclei were isolated using a nuclear extraction buffer, and chromatin was sheared by sonication to fragment sizes of 200–500 bp. Immunoprecipitation was carried out with a Flag‐specific antibody (AE092, ABclonal) to enrich DNA fragments bound to the target protein. Finally, the enriched DNA fragments corresponding to downstream target genes were quantified by ChIP‐qPCR, and enrichment factors were calculated, with three biological replicates and three technical replicates.

### 4.15 Dual‐Luciferase Reporter Assay

The full‐length CDS of *PagMYBR005* and *PagMYBR028* was cloned into the pGreenII 62‐SK vector to generate the effector construct pGreenII‐PagMYBR005/028‐62SK. Meanwhile, a 2000 bp promoter sequence of the *PagGRF12b* was inserted into the pGreenII0800‐LUC vector to drive the luciferase (LUC) reporter gene as a reporter construct. All recombinant plasmids were individually transformed into *Agrobacterium* strain GV3101 (pSoup).

For transient expression assays, equal volumes of agrobacterial cultures carrying the effector and reporter constructs were mixed and infiltrated into the abaxial side of tobacco leaves. The plants were then incubated for 2–3 days to promote transient expression. LUC fluorescence signals were captured using a Tanon‐5200 live‐cell fluorescence imaging system (Shanghai, China). And quantitative LUC activity was detected using Dual‐Luciferase Reporter Assay System (E1910, Promega). Firefly luciferase activity was normalized to the corresponding *Renilla* luciferase activity for each sample. All experiments included three biological replicates and three technical replicates.

## Author Contributions

G.F., G.Q. and T.J. conceived and designed the research. G.F., J.J., Z.S. and Y.Q. performed experiments and wrote the original draft. Z.C., T.J., G.F. and J.J. contributed to revising and providing critical comments on the manuscript. G.F., F.M. and Z.L. cultivates plant material. G.F. and Z.C. contributed to design the review and to make the final corrections for the manuscript. All authors read and approved the final manuscript.

## Funding

This work was supported by National Key Research and Development Program of China (2021YFD2200203), the Fundamental Research Fund of State Key Laboratory of Tree Genetics and Breeding (TGBFRF202517).

## Conflicts of Interest

The authors declare no conflicts of interest.

## Supporting information


**Figure S1:** The sequence characteristics analysis of PagMYBR028 protein. (a) Phylogenetic tree of PagMYBR028 homologues from *Populus alba* × *P. glandulosa* and other species. (b) Amino acid sequence alignment of PagMYBR028 and other homologues proteins in other species. The black line represents the MYB domain.
**Figure S2:** Detection of DNA and RNA levels in *PagMYBR028‐*transgenic lines. (a) DNA detection of *PagMYBR028‐*overexpression lines; (b) RT‐qPCR analysis of *PagMYBR028* expression in *PagMYBR028‐*overexpression lines; (c, d) DNA detection of *PagMYBR028*‐RNAi lines; (e) RT‐qPCR analysis of *PagMYBR028* expression in *PagMYBR028*‐RNAi lines. Data in (b, e) are presented as mean ± SD (*n* = 3). Asterisks indicate significant differences from the WT (Student's *t*‐test; *p < 0.05, **p < 0.01).
**Figure S3:** Phenotypic analysis of *PagMYBR028*‐transgenic plants and WT. (a) Plant height measurement. (b) Ground diameter measurement. Data are presented as mea*n* ± SD (*n* ≥ 15). Asterisks indicate significant differences from the WT (Student's *t*‐test; **p < 0.01).
**Figure S4:** Expression levels of cell expansion‐related (*EXPA3a*, *EXPA3b*) and cell cycle‐related (*CYCB1;1a*, *CYCB1;1b*) genes in WT and *PagMYBR028*‐transgenic poplar leaves. Data are presented as mea*n* ± SD (*n* ≥ 3). Asterisks indicate significant differences from the WT (Student's *t*‐test; **p < 0.01).
**Figure S5:** Xylem cell morphology in WT and *PagMYBR028*‐transgenic poplar. Representative cross‐sectional images of xylem cells from different stem nodes. Scale bar = 200 μm.
**Figure S6:** Expression of lignin biosynthesis‐related genes in WT and *PagMYBR028‐*transgenic poplar. Data are presented as mea*n* ± SD (*n* ≥ 3). Asterisks indicate significant differences from WT (Student's *t*‐test; *p < 0.05, **p < 0.01).
**Figure S7:** Amino acid sequence alignment between PagMYBR005 and PtrMYBR005.
**Figure S8:** Detection of DNA and RNA levels in *PagMYBR005‐*transgenic lines. (a) DNA detection of *PagMYBR005‐*overexpression lines; (b) RT‐qPCR analysis of *PagMYBR005* expression in *PagMYBR005‐*overexpression lines; (c, d) DNA detection of *PagMYBR005*‐RNAi lines; (e) RT‐qPCR analysis of *PagMYBR005* expression in *PagMYBR005*‐RNAi lines. Data in (b, e) are presented as mean ± SD (*n* = 3). Asterisks indicate significant differences from WT (Student's *t*‐test; *p < 0.05, **p < 0.01).
**Figure S9:** Phenotypic characterization of 3‐month‐old *PagMYBR005*‐transgenic lines and WT. (a) Plant height measurement. (b) Ground diameter measurement. Data are presented as mea*n* ± SD (*n* ≥ 15). Asterisks indicate significant differences from WT (Student's *t*‐test, **p < 0.01).
**Figure S10:** Expression levels of cell expansion‐related (*EXPA3a*, *EXPA3b*) and cell cycle‐related (*CYCB1;1a*, *CYCB1;1b*) genes in WT and *PagMYBR005*‐transgenic poplar leaves. Data are presented as mea*n* ± SD (*n* ≥ 3). Asterisks indicate significant differences from the WT (Student's *t*‐test, *p < 0.05, **p < 0.01).
**Figure S11:** Xylem cell morphology in WT and *PagMYBR005*‐transgenic poplar. Representative cross‐sectional images of xylem cells from different stem nodes. Scale bar = 200 μm.
**Figure S12:** Expression of lignin biosynthesis‐related genes in WT and *PagMYBR005‐*transgenic poplar. Data are presented as mea*n* ± SD (*n* ≥ 3). Asterisks indicate significant differences from WT (Student's *t*‐test; *p < 0.05, **p < 0.01).
**Figure S13:** Transcriptome profiling of *PagMYBR028*‐overexpressing poplar. (a) Volcano plot of differentially expressed genes (DEGs) in the *PagMYBR028*‐overexpression lines compared to WT. (b) KEGG pathway enrichment analysis of the identified DEGs.
**Figure S14:** Metabolomic profiling of *PagMYBR028*‐overexpressing poplar. (a) Volcano plot of differential metabolites in the *PagMYBR028*‐overexpression lines compared to WT. (b) KEGG pathway enrichment analysis of the identified differential metabolites.
**Figure S15:** RT‐qPCR analysis of *PagGRF12b* expression in *PagGRF12b*‐overexpression lines. Data are presented as mean ± SD (*n* = 3). Asterisks indicate significant differences from WT (Student's *t*‐test; **p < 0.01).
**Figure S16:** Xylem cell morphology in WT and *PagGRF12b*‐overexpression poplar. Representative cross‐sectional images of xylem cells from different stem nodes. Scale bar = 200 μm.

**Table S1:** Expression list of 152 MYB‐related transcription factors in different tissues in 84 K Poplar.
**Table S2:** List of Differential metabolite information.
**Table S3:** Expression list of genes in the phenylpropane biosynthesis pathway.
**Table S4:** Differential expression transcription factors information in paper.
**Table S5:** List of primers used in this paper.

## Data Availability

The Illumina RNA‐seq raw data from this study are available in NCBI (https://www.ncbi.nlm.nih.gov/) under accession numbers PRJNA736374 (for various tissues expression, including phloem‐cambium regions, xylem and shoot apical meristem) and PRJNA1356479 (for debarked stem tissues of WT and *PagMYBR028*‐OE poplar).
