# Supplementary material for: A MYB‐Related Transcriptional Module Cooperatively Represses Xylem Development in Poplar
Source: Plant Biotechnol J. 2026 Aug 21:10.1111/pbi.70745. Online ahead of print. doi: 10.1111/pbi.70745 (PMC13494651; doi:10.1111/pbi.70745)
Supplement: Supplementary file 1 [file PBI-9999-0-s001.docx]

**Supporting information**

**Figure S1 The sequence characteristics analysis of PagMYBR028** **protein**. (a) Phylogenetic tree of PagMYBR028 homologs from *Populus alba* × *P. glandulosa* and other species. (b) Amino acid sequence alignment of PagMYBR028 and other homologs proteins in other species. The black line represents the MYB domain.

**Figure S2 Detection of DNA and RNA levels in *PagMYBR028-*transgenic lines.** (a) DNA detection of *PagMYBR028-*overexpression lines; (b) RT-qPCR analysis of *PagMYBR028* expression in *PagMYBR028-*overexpression lines; (c, d) DNA detection of *PagMYBR028*-RNAi lines; (e) RT-qPCR analysis of *PagMYBR028* expression in *PagMYBR028*-RNAi lines. Data in (b, e) are presented as mean ± SD (n = 3). Asterisks indicate significant differences from the WT (Student's *t-test*; * *p* < 0.05, ** *p* < 0.01).

**Figure S3. Phenotypic analysis of *PagMYBR028*-transgenic plants and WT.** (a) Plant height measurement. (b) Ground diameter measurement. Data are presented as mean ± SD (n ≥ 15). Asterisks indicate significant differences from the WT (Student's *t-test*; ** *p* < 0.01).

**Figure S4** Expression levels of cell expansion-related (*EXPA3a*, *EXPA3b*) and cell cycle-related (*CYCB1;1a*, *CYCB1;1b*) genes in WT and *PagMYBR028*-transgenic poplar leaves. Data are presented as mean ± SD (n ≥ 3). Asterisks indicate significant differences from the WT (Student's *t-test*; ** *p* < 0.01).

**Figure S5. Xylem cell morphology in WT and *PagMYBR028*-transgenic poplar.** Representative cross-sectional images of xylem cells from different stem nodes. Scale bar = 200 μm.

**Figure S6** Expression of lignin biosynthesis-related genes in WT and *PagMYBR028-*transgenic poplar. Data are presented as mean ± SD (n ≥ 3). Asterisks indicate significant differences from WT (Student's *t-test*; * *p* < 0.05, ** *p* < 0.01).

**Figure S7.** Amino acid sequence alignment between PagMYBR005 and PtrMYBR005.

**Figure S8 Detection of DNA and RNA levels in *PagMYBR005-*transgenic lines.** (a) DNA detection of *PagMYBR005-*overexpression lines; (b) RT-qPCR analysis of *PagMYBR005* expression in *PagMYBR005-*overexpression lines; (c, d) DNA detection of *PagMYBR005*-RNAi lines; (e) RT-qPCR analysis of *PagMYBR005* expression in *PagMYBR005*-RNAi lines. Data in (b, e) are presented as mean ± SD (n = 3). Asterisks indicate significant differences from WT (Student's *t-test*; * *p* < 0.05, ** *p* < 0.01).

**Figure S9. Phenotypic characterization of 3-month-old *PagMYBR005*-transgenic lines and WT.** (a) Plant height measurement. (b) Ground diameter measurement. Data are presented as mean ± SD (n ≥ 15). Asterisks indicate significant differences from WT (Student's *t-test*, ** *p* < 0.01).

**Figure S10** Expression levels of cell expansion-related (*EXPA3a*, *EXPA3b*) and cell cycle-related (*CYCB1;1a*, *CYCB1;1b*) genes in WT and *PagMYBR005*-transgenic poplar leaves. Data are presented as mean ± SD (n ≥ 3). Asterisks indicate significant differences from the WT (Student's *t-test*, * *p* < 0.05, ** *p* < 0.01).

**Figure S11. Xylem cell morphology in WT and *PagMYBR005*-transgenic poplar.** Representative cross-sectional images of xylem cells from different stem nodes. Scale bar = 200 μm.

**Figure S12** Expression of lignin biosynthesis-related genes in WT and *PagMYBR005-*transgenic poplar. Data are presented as mean ± SD (n ≥ 3). Asterisks indicate significant differences from WT (Student's *t-test*; * *p* < 0.05, ** *p* < 0.01).

**Figure S13. Transcriptome profiling of *PagMYBR028*-overexpressing poplar.** (a) Volcano plot of differentially expressed genes (DEGs) in the *PagMYBR028*-overexpression lines compared to WT. (b) KEGG pathway enrichment analysis of the identified DEGs.

**Figure S14. Metabolomic profiling of *PagMYBR028*-overexpressing poplar.** (a) Volcano plot of differential metabolites in the *PagMYBR028*-overexpression lines compared to WT. (b) KEGG pathway enrichment analysis of the identified differential metabolites.

**Figure S15.** RT-qPCR analysis of *PagGRF12b* expression in *PagGRF12b*-overexpression lines. Data are presented as mean ± SD (n = 3). Asterisks indicate significant differences from WT (Student's *t-test*; ** *p* < 0.01).

**Figure S16. Xylem cell morphology in WT and *PagGRF12b*-overexpression poplar.** Representative cross-sectional images of xylem cells from different stem nodes. Scale bar = 200 μm.
